# Global Dialysis Perspective: Lebanon

**DOI:** 10.34067/KID.0000000000000207

**Published:** 2023-07-07

**Authors:** Mabel Aoun, Sahar Koubar

**Affiliations:** 1 Faculty of Medicine, Saint-Joseph University of Beirut, Beirut, Lebanon; 2 Faculty of Medicine, University of Minnesota, Minneapolis, Minnesota

**Keywords:** anemia, chronic hemodialysis, CKD, dialysis, ESKD, epidemiology and outcomes, hypertension, polycystic kidney disease, renal pathology, vascular calcification

## Lebanese Population and Health Care System

**Figure 1.**
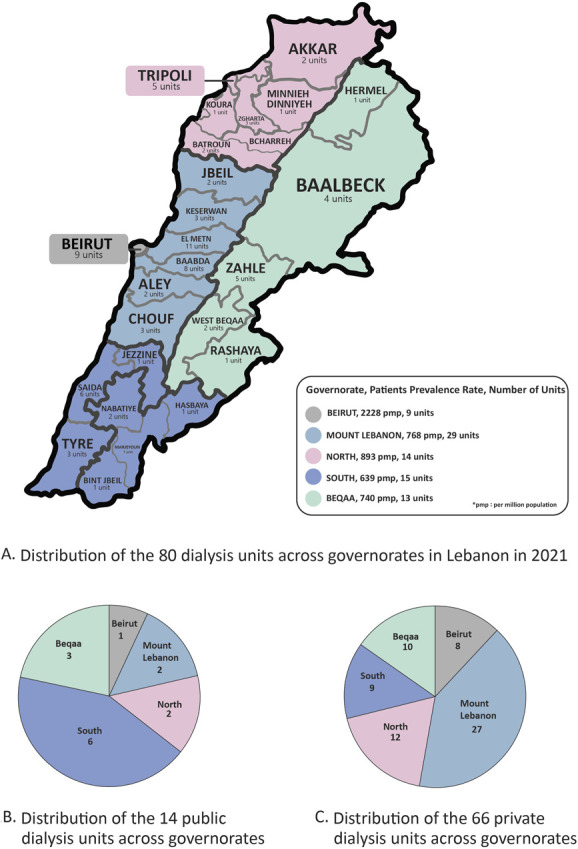
**Distribution of HD across governorates in 2021.** (A) Distribution of the 80 dialysis units across governorates in Lebanon in 2021. (B) Distribution of the 14 public dialysis units across governorates. (C) Distribution of the 66 private dialysis units across governorates. HD, hemodialysis.

Lebanon is a 10,452 square kilometer Middle Eastern country that is known for its cedar trees, multireligious cohabitation, political turmoil, world highest ratio of refugee to native population, and lately one of the worst economic crises in the world history. Lebanon is divided into nine governorates that can be geographically reassembled into the following five major ones: Beirut, Mount Lebanon, North, South, and Beqaa (Figure 1). Owing to political reasons, Lebanon has not held a proper population census since 1932. According to the United Nations, Lebanon's population was estimated at 5,631,000 inhabitants in 2021 including Lebanese citizens and refugees.^1^ However, the latest estimate of Lebanese citizens reported by the central administration statistics of the Lebanese council of ministers in 2020 was 4,842,000,^2^ and the number of refugees reported by the United Nations High Commissioner for Refugees fact sheets was 1.5 million.^3^

The Lebanese health care system is known to be complex and pluralistic.^4^ Although 80% of hospitals in Lebanon are private, health care services are largely covered by public third-party payers. These public payers include the Lebanese Ministry of Public Health (MoPH), National Social Security Fund (NSSF), Civil Servants Cooperative, Army/Security institutions, and Municipalities. These public institutions only cover Lebanese patients, whereas refugees are supported by the United Nations High Commissioner for Refugees and Non-Governmental Organizations (NGOs). As a result, refugees and their data are not officially part of the Lebanese health care system.^3,5^ The MoPH is the major contributor in the public sector, and it is the country's highest health authority, enforcing health regulations by releasing policies and decrees. From the year 2005 onward, a financial ceiling was set in contracts between hospitals and MoPH; this ceiling limited the number of hospitalizations and chronic dialysis patients covered by the MoPH in each hospital.

## The Economic and Health Care Crisis

The era after the end of the civil war in 1990 witnessed significant economic growth. Based on the World Bank economic indicators, the Lebanese gross domestic product *per capita* increased from <1000 $US in 1990 to 9226 $US in 2018.^6^ During the period 1995–2022, Lebanon was ranked as an upper middle-income country. Some of Lebanon's health indicators reported by the United Nation were close to those of developed countries.^1^ Life expectancy for example was estimated at 79.7 years in 2017.^1^ The health sector was allocated 3% of the governmental budget, and this budget ensured universal coverage of dialysis, heart surgeries, and chemotherapy. In addition, half of the Lebanese population was entitled to MoPH coverage because of lack of social, military, or private insurance.

However, since the end of 2019, Lebanon has experienced a massive devaluation of its currency, the Lebanese pound (LBP), leading to surging inflation rates and pushing half of the population below poverty levels. In July 2022, the World Bank downgraded Lebanon to a low middle-income country.^6^ The Lebanese MoPH and other public institutions attempted to increase the reimbursement of dialysis, but owing to the ongoing currency devaluation, the reimbursement fees were not sufficient to cover the dialysis disposables. Consequently, a significant number of patients started to pay out of pocket for their dialysis treatment. This review will provide information on the costs and standards of dialysis in times of economic stability and in the aftermath of inflation and economic crisis.

## Dialysis Distribution

### Data Sources

Despite the strong health care system and the high quality of health care services that Lebanon offered before the economic crisis, health authorities failed to build national registries for different diseases in Lebanon. The data included in this review are collected from conferences,^7^ COVID data in hemodialysis (HD) units published on the MoPH website,^8^ and a few publications.^9–15^ Table 1 summarizes the characteristics of dialysis in Lebanon.

**Table 1.** Characteristics of dialysis in Lebanon

No. of dialysis patients	4400 (prevalent patients in 2021–2022) 191 pmp (incident patients last assessed by MoPH in 2015)
No. of patients on PD	210 (prevalent patients in 2021–2022)
No. of kidney transplant patients	Close to 1000
Dialysis sessions: covered by insurance versus private-paying patients	All sessions covered by the public third-party payers (MoPH, NSSF, CSC, military)
Dialysis units: hospital-based or freestanding	All hospital-based
Dialysis units: economic model	Nonprofit in public and private hospitals
Mean reimbursement per dialysis session (in US$)	$126 before the crisis (2014–2021) $66 amid the crisis (early 2023)
Dialysis delivery staff	Only dialysis nurses
Typical patient-to-RN ratio in the dialysis unit	4:1 (recommended by Lebanese health authorities, not applied in all units)
Average length of a dialysis session	240 min (recommended by Lebanese health authorities, not applied in all units)
No. of times per month patients are seen by a nephrologist during dialysis sessions	Every dialysis session
Proportion of prevalent HD patients using an AVF, AVG, and CVC	No registry data; 11%–38% of patients using a CVC, based on two studies and four units

MoPH, Ministry of Public Health; PD, peritoneal dialysis; NSSF, National Social Security Fund; CSC, Civil Servants Cooperative; RN, registered nurse; HD, hemodialysis; AVF, arteriovenous fistula; AVG is arteriovenous graft CVC, central venous catheter.

### Home Dialysis Modalities: Peritoneal Dialysis and Home HD

Peritoneal dialysis (PD) is the main home-based modality offered in Lebanon. MoPH started covering the cost of PD solutions in 1997. However, patients pay out of pocket the physician's fees and rental fees for the cycler if they opt for using automated PD. One significant challenge associated with automated PD is the unreliable electricity supply in Lebanon. To ensure uninterrupted functionality of the cycler amid frequent power outages and the high costs of generators, patients who choose this technique must invest in an expensive uninterruptible power source. As of 2015, the reported number of PD patients was 150, and by late 2022, the estimate increased to approximately 210 (based on personal communication). For PD patients who are not eligible for NSSF coverage, the MoPH provides free erythropoietin treatment.

Home HD is exceptional in Lebanon, with only two patients currently using this modality (personal communication). Home HD is not reimbursed and must be fully paid out of pocket by the patients.

### Hospital-Based HD

**Figure 2.**
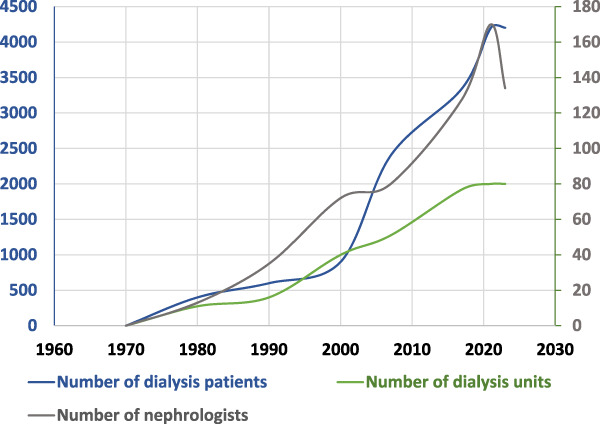
Growing number of dialysis patients, units, and nephrologists between 1970 and 2022.

In Lebanon, all HD services are exclusively provided within hospital-based units. According to Lebanese regulations, dialysis centers are not allowed to operate outside of hospitals. The introduction of HD treatment in Lebanon dates back to 1969.^7^ All dialysis sessions are scheduled during the daytime and evening, and currently, there is no provision of nocturnal HD in the country. Figure 2 depicts the number of nephrologists, dialysis units, and HD patients over time. The number of nephrologists working in dialysis units increased from 13 in 1980, to 132 in 2015 to more than 160 in 2021; it then decreased between 2021 and 2022 after the nephrologists' exodus instigated by the economic crisis.^7,9^ Dialysis units grew in number to a peak of 80 facilities in 2021.^8^ The total number of HD patients increased from 500 in the 80s to 2400 in 2007, 3350 in 2015, and 4202 in 2021.

### Prevalence and Incidence Rates of Dialysis

Between 2014 and 2015, the estimated prevalence and incidence rates of ESKD requiring dialysis were 777 and 191 per million (pm) population, respectively. Specifically, the prevalence rates for HD and PD were estimated at 744 and 33 pmp, respectively, while the incidence rates for HD and PD were 187 and 4 pmp, respectively.^9^ In 2021, the MoPH reported a total of 4202 HD patients^8^ with a prevalence rate of HD estimated at 875 pmp across all governorates. The distribution of HD units and patients across governorates in 2021 is depicted in Figure 1.

## Financing and Reimbursement

### HD Sessions

Since 1979, as dictated by a presidential decree, all Lebanese citizens who suffer from ESKD have been provided with comprehensive coverage of HD sessions by the NSSF and MoPH.^7^ The reimbursement for the HD session is structured as a combined payment or bundled fee that includes fees for both the hospital and the physician (Supplemental Table 1). This bundled payment encompasses the direct medical costs associated with a session but excludes transportation and opportunity costs (Supplemental Table 2). It is estimated that hospitals and physicians typically experience a delay of approximately 12 months before receiving payment from the various third-party payers.^13^

### Chronic Medications' Coverage for Dialysis Patients

The MoPH provides free chronic medications to half of the Lebanese patients on dialysis, mainly those who are not registered at the NSSF. The list of these medications includes alfacalcidol, iron sucrose, sevelamer, lanthanum, and cinacalcet. Patients who benefit from NSSF coverage are reimbursed 90% of medications' costs after a 6-month to 12-month delay. Patients covered by Army/Security institutions have free access to all medications.

### Cost of Illness of Dialysis

**Figure 3.**
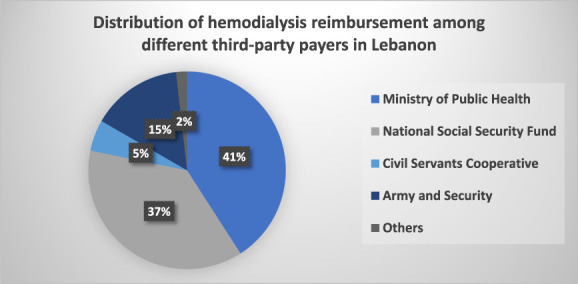
**Distribution of HD reimbursement among different payers.** HD, hemodialysis.

Figure 3 illustrates the breakdown of HD reimbursement across all third-party payers, excluding private insurances because they do not cover dialysis. According to a study conducted in 2020, taking into account the old exchange rate of $1=1500 LBP, the estimated annual median societal cost per patient for HD was 27,818 $US (equivalent to 42,144,500 LBP) while for PD it was 28,595 $US (equivalent to 43,322,488 LBP), respectively.^10^ In the case of HD, the expenses primarily originated from physicians, nurses, and treatment fees, whereas in PD, the main cost driver was the specific technique used. In the same study, from the third-party payer perspective, the estimated annual cost per patient for HD was 23,380 $US (equivalent to 35,420,500 LBP), and for PD, it was 26,033 $US (equivalent to 39,440,000 LBP), respectively.^10^ These findings result in a total national annual cost of 98,195,445 $US for HD before the inflation surge. It is worth noting that, at the initial phases of the crisis between 2019 and 2020, medical supplies, laboratory kits, and medications were still subsidized by the government while the salaries of nurses in most hospitals remained unchanged.

## Quality of Care

### Regulations

Most of the quality-of-care aspects in HD in Lebanon were recommended by the 2014 MoPH decree and its corresponding guideline**.**

### Human Resources

Based on MoPH recommendations, nephrologists are required to personally attend to patients undergoing HD during each session. In addition, the regulations state that a nephrologist should not work in more than one unit and should not care for more than 40 patients on dialysis.^9^ However, a survey of nephrologists' satisfaction revealed that some of them would prefer to care for a larger number of patients beyond the recommended limit of 40.^13^ Furthermore, it is recommended to maintain a staffing ratio of one registered nurse for every four dialysis patients and one nurse's aide for every six patients.^9^

### Water Quality and High-Flux Membranes

In 2014, dialysis units embarked on the installation of an upgraded water treatment system, enabling the provision of ultrapure fluids.^9^ Owing to the limited number of companies capable of supplying the necessary double reverse osmosis and endotoxin filters, it took a period of 5 years to implement this high-quality treatment across all 80 dialysis facilities. Ultrapure water was defined as follows: maximum allowable levels of endotoxin <0.03 EU/mL and total viable microbial count <0.1 colony-forming unit/ml, to be tested monthly with total chlorine <0.1 mg/L and total dissolved solids <10 ppm.

### Length of Session and Frequency

Back in 1997, the MoPH provided coverage for only one-third of patients to receive three sessions per week while the remaining two-thirds were limited to twice per week.^7^ However, over time, a higher percentage of patients gradually gained access to thrice-weekly dialysis. The 2014 MoPH regulations recommended three sessions per week, with each session lasting 4 hours^9^ Following this guideline, most of the units adopted the recommended treatment frequency and duration.

### Laboratory Tests' Monitoring

The MoPH set the schedule for laboratory tests monitoring in all dialysis units in 2014 as summarized in Supplemental Table 3. This schedule was amended after the economic crisis to reduce costs.

### Vascular Access

There are limited available data on HD vascular access in Lebanon. Two published studies provide some insights. According to these studies, the utilization of tunneled central venous catheters (CVCs) for HD ranged between 11.2 and 38%.^11,12^ The first study examined 218 chronic HD patients at a major university hospital center between 2008 and 2015.^11^ The patients' age at dialysis initiation ranged from 64 to 71 years with 53.2% of them having diabetes. Arteriovenous fistula was the predominant vascular access method, accounting for 62% of cases.^11^ Another study involved centers located in three different Lebanese districts and included 214 HD patients between 2012 and 2020. The mean age of the patients was 68 years, and 51.4% had diabetes. In this study, only 11.2% of the patients had tunneled CVCs for chronic HD.^12^ It is worth noting that all the HD centers included in these two studies were privately run nongovernmental institutions.

## Refugees on Dialysis in Lebanon

Palestinian refugees have been residing in Lebanon for over 70 years, while Syrian refugees started entering Lebanon after the onset of the Syrian war in 2011.^5^ A large dialysis unit located in the South of Lebanon has been providing chronic dialysis services to Palestinians. The costs associated with their treatment are covered by NGOs. This unit does not receive reimbursement from the Lebanese MoPH or other public third-party payers; thus, it does not report to the MoPH. As for the Syrian refugees, 200–250 have received dialysis in Lebanon over the last decade.^5^ Initially, the influx of these patients was disorganized, with some seeking services in private hospitals and paying out of pocket for their dialysis sessions, while others received free dialysis treatment in public hospitals. However, dialysis services were later organized, and Syrian refugees in need of dialysis were redirected to specific Lebanese dialysis units that received reimbursement through multiple NGOs.^5^ Unfortunately, no data are available regarding the quality of treatment provided to these patients.

## Dialysis after the Economic Collapse

Amid the ongoing economic crisis, the government subsidy's rate for dialysis filters was set at 85%, while the remaining costs had to be covered by the bundled fee reimbursed by third-party payers. However, owing to mounting inflation, the bundled fee was no longer sufficient to cover expenses such as medications, electricity, and human resources. Each dialysis unit responded differently to rising inflation and changing circumstances. Some units decreased the laboratory tests and the number of weekly sessions.^15^ In almost all units, the decrease in the number of weekly sessions was necessitated by high fuel prices, which rendered transportation costs unaffordable for patients. Some units tried to sustain the dialysis services and treatment of patients by accepting donations from NGOs and private donors. The availability of relevant medications such as alfacalcidol, cinacalcet, vitamin B, and heparin was inconsistent throughout the country. To cover the increased costs of dialysis sessions, some units placed a charge that the patient had to pay out of pocket each session or each month to cover part or whole of the dialysis session cost. A significant number of nephrologists left the country, adding a burden on the remaining nephrologists who were forced to handle a higher number of patients than had previously been permitted. The MoPH recently issued an amendment of the laboratory monitoring schedule after months of unavailable kits for parathyroid hormone and hepatitis serology testing. The national response to the crisis was an increase in the amount of the reimbursed bundled fee (Supplemental Table 1).

## Limitations of This Review

We recognize two limitations primarily arising from the limited availability of published data. First, amid the absence of a national dialysis registry in Lebanon, most of the information was gathered from conferences, research articles, and personal communications. Second, when calculating prevalence and incidence rates, we had to depend on a rough estimate of the Lebanese population because no national population census was available. Moreover, the ongoing economic crisis has led to frequent fluctuations in the exchange rate, which adds complexity to reporting costs.

## Future Challenges

In light of the ongoing currency devaluation and the exodus of nephrologists and nurses from the country, dialysis runs two major risks, the first is the potential compromise of treatment quality and the second one is the unaffordable societal cost of dialysis. It is evident that the economic collapse led to a catastrophic societal chasm in this small Middle Eastern country and could result in an unequal distribution of dialysis services between the wealthy and the poor. Unfortunately, there is no national registry to address this challenge. Therefore, health authorities and scientific societies are strongly urged to invest tremendous efforts into data collection and reporting because health policies are only relevant if evidence-based and contextualized to local needs.

## Disclosures

M. Aoun reports the following consultancy: APIS Health Consulting Group. The remaining author has nothing to disclose.

## Supplementary Material

## Supplemental Material

Supplemental Table 1. Reimbursed bundled fee per session across years and by all third-party payers.

Supplemental Table 2. Estimation of dialysis costs covered by the dialysis bundled fee before the inflation.

Supplemental Table 3. Frequency of routine laboratory tests in hemodialysis.
