# Supplementary material for: Global Dialysis Perspective: Lebanon
Source: Kidney360. 2023 Jul 7;4(9):e1308–13. doi: 10.34067/KID.0000000000000207 (PMC10547222; doi:10.34067/KID.0000000000000207)
Supplement: Supplementary file 1 [file kidney360-4-e1308-s001.pdf]

Table S1. Reimbursed bundled fee per session across years and by all third-party payers

	Reimbursed bundled fee
**Hospital**	
**Two decades prior to 2014**	72 $US (110,000 LBP)
**2014- Jan 2020**	101 $US (154,000 LBP)
START OF INFLATION	
**2020-2021**	Varied between 38 $US and 19 $US (154,000 LBP)
**2021 (Sept-Dec)**	18 $US (500,000 LBP)
**2022 (April)**	37 $US (1,000,000 LBP) becomes 10 $US if paid in 2023
**2023 (Jan-Feb)**	25 $US (2,500,000 LBP)
**2023 April**	52 $US (5,240,000 LBP)
**Physician**	
**Two decades prior to 2014**	16 $US (25,000 LBP)
**2014- Jan 2020**	25 $US (37,500 LBP)
START OF INFLATION	
**2020- 2021**	Varied between 9 $US and 4.5 $US (37,500 LBP)
**2021 (Sept-Dec)**	2.8 $US (75,000 LBP)
**2022 (April)**	7.4 $US (200,000 LBP) becomes 2 $US if paid in 2023
**2023 (Jan-Feb)**	5 $US (500,000 LBP)
**2023 April**	14 $US (1,400,000 LBP)

Note. Third-party payers are MoPH, NSSF, CSC, ISF, Army and General Security, Municipalities. Private insurances do not cover chronic dialysis.
The exchange rates of $US: 1,515 LBP before 2020; 18,000 LBP in Sept 2021; 27,000 LBP in Dec 2021 and Apr 2022; 100,000 LBP in 2023. The delay in payments with the progressive currency devaluation will lead to smaller reimbursement fees.

Table S2. Estimation of dialysis costs covered by the dialysis bundled fee before the inflation

**Direct costs covered by the total bundled fee per session:**						
Total amount of hemodialysis bundled fee	Dialysis kit: * Filter * Bicarbonate cartridge * Endotoxin filter * Tubes * Needles	Medications: * Heparin * EPO	Lab tests: * Routine blood tests * Water endotoxin * Chlorine tests	* Water treatment (double reverse osmosis), * Electricity, * Laundry, * Lunch, * Infrastructure, * Maintenance, depreciation	Nurses	Physicians
**125 $US**	**25 $US**	**15 $US**	**15 $US**	**30 $US**	**15 $US**	**25 $US**

Note. Costs such as transportation or opportunity costs are not reimbursed

Table S3. Frequency of routine laboratory tests in hemodialysis

	2014 Schedule	2022 Schedule (crisis-related)
**CBC**	Once per month	Once per month
**Potassium pre-dialysis**	Once per month	Once per month
**Creatinine, sodium, chloride, calcium, phosphate, bicarbonate, aspartate transaminase (AST)**	Once per month	Every two months
**BUN pre- and post-dialysis**	Once per month	Every three months
**Alkaline phosphatase**	Every 4 months	Every 6 months
**Albumin/Protein**	Every 4 months	Every 6 months
**Ferritin, TSAT, PTH, magnesium**	Every 4 months	Every 6 months
**Hbs Antigen**	Every 4 months	Every 4 months for non-immunized and every 6 months for immunized
**Hbs Antibodies**	Every 4 months	Every year
**HCV serology**	Every 4 months	Every year
**Lipid panel, HIV serology, PPD**	Every year	Every year
